# 3-(4-Fluoro­phenyl­sulfin­yl)-2-methyl-1-benzofuran

**DOI:** 10.1107/S1600536810004411

**Published:** 2010-02-10

**Authors:** Hong Dae Choi, Pil Ja Seo, Byeng Wha Son, Uk Lee

**Affiliations:** a Department of Chemistry, Dongeui University, San 24 Kaya-dong Busanjin-gu, Busan 614-714, Republic of Korea; b Department of Chemistry, Pukyong National University, 599-1 Daeyeon 3-dong, Nam-gu, Busan 608-737, Republic of Korea

## Abstract

In the title compound, C_15_H_11_FO_2_S, the O atom and the 4-fluoro­phenyl group of the 4-fluoro­phenyl­sulfinyl substituent lie on opposite sides of the plane of the benzofuran fragment; the 4-fluoro­phenyl ring is almost perpendicular to this plane [dihedral angle = 89.59 (5)°]. Inter­molecular C—H⋯F and C—H⋯O hydrogen bonds link the mol­ecules together in the crystal structure.

## Related literature

For the crystal structures of similar 2-methyl-3-phenyl­sulfinyl-1-benzofuran derivatives, see: Choi *et al.* (2008**a* ▶,*b* ▶,c* ▶). For the pharmacological activity of benzofuran compounds, see: Aslam *et al.* (2006 ▶); Galal *et al.* (2009 ▶); Khan *et al.* (2005 ▶). For natural products with benzofuran rings, see: Akgul & Anil (2003 ▶); Soekamto *et al.* (2003 ▶).
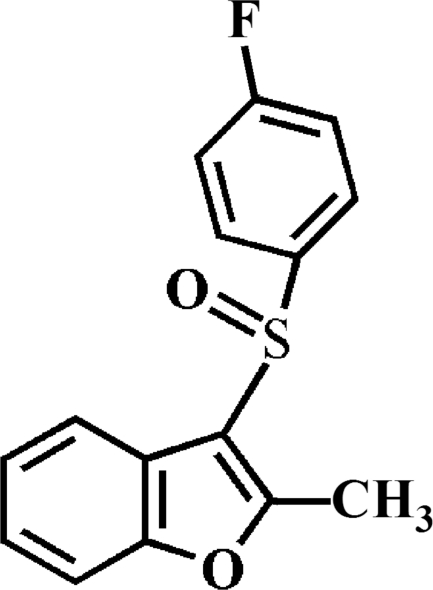


## Experimental

### 

#### Crystal data

C_15_H_11_FO_2_S
*M* *_r_* = 274.30
Orthorhombic, 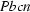

*a* = 14.992 (1) Å
*b* = 10.4661 (8) Å
*c* = 16.008 (1) Å
*V* = 2511.8 (3) Å^3^
*Z* = 8
Mo *K*α radiation
μ = 0.26 mm^−1^
*T* = 173 K
0.50 × 0.50 × 0.25 mm

#### Data collection

Bruker SMART APEXII CCD diffractometer
Absorption correction: multi-scan (*SADABS*; Bruker, 2009 ▶) *T*_min_ = 0.880, *T*_max_ = 0.937
14681 measured reflections
2878 independent reflections
2315 reflections with *I* > 2σ(*I*)
*R*_int_ = 0.026

#### Refinement

*R*[*F* ^2^ > 2σ(*F* ^2^)] = 0.035
*wR*(*F* ^2^) = 0.101
*S* = 1.10
2878 reflections
173 parameters
H-atom parameters constrained
Δρ_max_ = 0.29 e Å^−3^
Δρ_min_ = −0.32 e Å^−3^

### 

Data collection: *APEX2* (Bruker, 2009 ▶); cell refinement: *SAINT* (Bruker, 2009 ▶); data reduction: *SAINT*; program(s) used to solve structure: *SHELXS97* (Sheldrick, 2008 ▶); program(s) used to refine structure: *SHELXL97* (Sheldrick, 2008 ▶); molecular graphics: *ORTEP-3* (Farrugia, 1997 ▶) and *DIAMOND* (Brandenburg, 1998 ▶); software used to prepare material for publication: *SHELXL97*.

## Supplementary Material

Crystal structure: contains datablocks I. DOI: 10.1107/S1600536810004411/bg2329sup1.cif

Structure factors: contains datablocks I. DOI: 10.1107/S1600536810004411/bg2329Isup2.hkl

Additional supplementary materials:  crystallographic information; 3D view; checkCIF report

## Figures and Tables

**Table 1:** Hydrogen-bond geometry (Å, °)

*D*—H⋯*A*	*D*—H	H⋯*A*	*D*⋯*A*	*D*—H⋯*A*
C6—H6⋯F^i^	0.93	2.52	3.346 (2)	148
C9—H9*B*⋯O2^ii^	0.96	2.41	3.189 (2)	138

Symmetry codes: (i) 
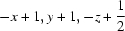
; (ii) 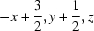.

**Fig. 1.**
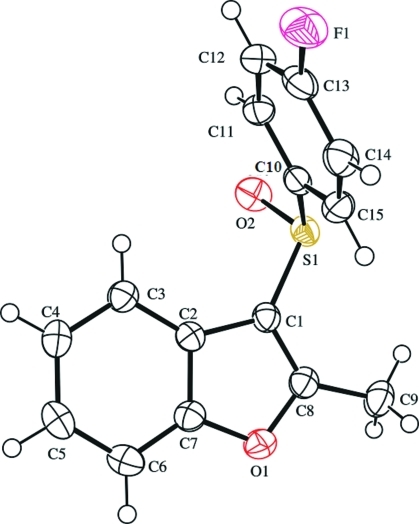
The molecular structure of the title compound with the atom numbering scheme. Displacement ellipsoids are drawn at the 50 % probability level. H atoms are presented as a small spheres of arbitrary radius.

**Fig. 2.**
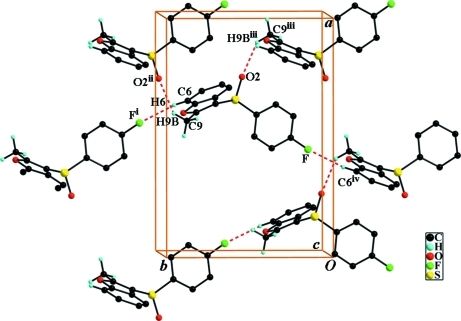
C—H···F and C—H···O interactions (dotted lines) in the crystal structure of the title compound. [Symmetry codes: i) - x + 1, y + 1, - z + 1/2; ii) - x + 3/2, y + 1/2, z; iii) - x + 3/2, y - 1/2, z; iv) - x + 1, y - 1, - z + 1/2.]

## supplementary crystallographic information

### Comment

Molecules containing benzofuran skeleton show significant pharmacological activities such as antifungal (Aslam *et al.*, 2006), antitumor and antiviral (Galal *et al.*, 2009), antimicrobial (Khan *et al.*, 2005) properties. These compounds are widely occurring in nature (Akgul & Anil, 2003; Soekamto *et al.*, 2003). As a part of our ongoing studies of the effect of side chain substituents on the solid state structures of 2-methyl-3-phenylsulfinyl-1-benzofuran analogues (Choi *et al.*, 2008*a,b,c*), we report the crystal structure of the title compound (Fig. 1).

The benzofuran unit is essentially planar, with a mean deviation of 0.012 (1) Å from the least-squares plane defined by the nine constituent atoms. The 4-fluorophenyl ring is almost perpendicular to the plane of the benzofuran fragment [89.59 (5)°] and is tilted slightly towards it. The crystal packing (Fig. 2) is stabilized by two different intermolecular  hydrogen bonds; the first one a C—H···F contact between the benzene H atom and the fluorine (Table 1, first entry ),  and the second, a C—H···O one between one methyl H atom and the oxygen of the S═O unit (Table 1, second entry).

### Experimental

77% 3-Chloroperoxybenzoic acid (359 mg, 1.6 mmol) was added in small portions to a stirred solution of 3-(4-fluorophenylsulfanyl)-2-methyl-1-benzofuran (387 mg, 1.5 mmol) in dichloromethane (40 mL) at 273 K. After being stirred at room temperature for 4h, the mixture was washed with saturated sodium bicarbonate solution and the organic layer was separated, dried over magnesium sulfate, filtered and concentrated in vacuum. The residue was purified by column chromatography (hexane-ethyl acetate, 1:1 v/v) to afford the title compound as a colorless solid [yield 78%, m.p. 415–416 K; *R*_f_ = 0.63 (hexane-ethyl acetate, 1:1 v/v)]. Single crystals suitable for X-ray diffraction were prepared by slow evaporation of a solution of the title compound in benzene at room temperature.

### Refinement

All H atoms were positioned geometrically and refined using a riding model, with C—H = 0.93 Å for aryl and 0.96 Å for methyl H atoms. *U*_iso_(H) = 1.2*U*_eq_(C) for aryl and 1.5*U*_eq_(C) for methyl H atoms.

### Crystal data

**Table:**

C_15_H_11_FO_2_S	*F*(000) = 1136
*M**_r_* = 274.30	*D*_x_ = 1.451 Mg m^−^^3^
Orthorhombic, *P**b**c**n*	Mo *K*α radiation, λ = 0.71073 Å
Hall symbol: -P 2n 2ab	Cell parameters from 6779 reflections
*a* = 14.992 (1) Å	θ = 2.4–27.5°
*b* = 10.4661 (8) Å	µ = 0.26 mm^−^^1^
*c* = 16.008 (1) Å	*T* = 173 K
*V* = 2511.8 (3) Å^3^	Block, colourless
*Z* = 8	0.50 × 0.50 × 0.25 mm

### Data collection

**Table:**

Bruker SMART APEXII CCD diffractometer	2878 independent reflections
Radiation source: Rotating Anode	2315 reflections with *I* > 2σ(*I*)
Bruker HELIOS graded multilayer optics	*R*_int_ = 0.026
Detector resolution: 10.0 pixels mm^-1^	θ_max_ = 27.5°, θ_min_ = 2.4°
φ and ω scans	*h* = −18→19
Absorption correction: multi-scan (*SADABS*; Bruker, 2009)	*k* = −13→13
*T*_min_ = 0.880, *T*_max_ = 0.937	*l* = −15→20
14681 measured reflections	

### Refinement

**Table:**

Refinement on *F*^2^	Primary atom site location: structure-invariant direct methods
Least-squares matrix: full	Secondary atom site location: difference Fourier map
*R*[*F*^2^ > 2σ(*F*^2^)] = 0.035	Hydrogen site location: difference Fourier map
*wR*(*F*^2^) = 0.101	H-atom parameters constrained
*S* = 1.10	*w* = 1/[σ^2^(*F*_o_^2^) + (0.0443*P*)^2^ + 1.1695*P*] where *P* = (*F*_o_^2^ + 2*F*_c_^2^)/3
2878 reflections	(Δ/σ)_max_ < 0.001
173 parameters	Δρ_max_ = 0.29 e Å^−^^3^
0 restraints	Δρ_min_ = −0.31 e Å^−^^3^

### Special details

**Table:**

Geometry. All esds (except the esd in the dihedral angle between two l.s. planes) are estimated using the full covariance matrix. The cell esds are taken into account individually in the estimation of esds in distances, angles and torsion angles; correlations between esds in cell parameters are only used when they are defined by crystal symmetry. An approximate (isotropic) treatment of cell esds is used for estimating esds involving l.s. planes.
Refinement. Refinement of F^2^ against ALL reflections. The weighted R-factor wR and goodness of fit S are based on F^2^, conventional R-factors R are based on F, with F set to zero for negative F^2^. The threshold expression of F^2^ > 2sigma(F^2^) is used only for calculating R-factors(gt) etc. and is not relevant to the choice of reflections for refinement. R-factors based on F^2^ are statistically about twice as large as those based on F, and R- factors based on ALL data will be even larger.

### Fractional atomic coordinates and isotropic or equivalent isotropic displacement parameters (Å^2^)

**Table:**

	*x*	*y*	*z*	*U*_iso_*/*U*_eq_	
S	0.65767 (3)	0.58401 (4)	0.06490 (3)	0.03017 (13)	
F	0.44627 (8)	0.14149 (11)	0.17017 (8)	0.0492 (3)	
O1	0.59892 (8)	0.88631 (11)	0.19868 (8)	0.0342 (3)	
O2	0.75317 (9)	0.54319 (13)	0.06877 (8)	0.0394 (3)	
C1	0.63870 (11)	0.69730 (16)	0.14353 (10)	0.0276 (3)	
C2	0.66131 (10)	0.69382 (15)	0.23170 (10)	0.0265 (3)	
C3	0.70180 (11)	0.60712 (17)	0.28570 (11)	0.0310 (4)	
H3	0.7212	0.5278	0.2668	0.037*	
C4	0.71236 (12)	0.64306 (18)	0.36876 (11)	0.0352 (4)	
H4	0.7384	0.5862	0.4062	0.042*	
C5	0.68456 (13)	0.76314 (19)	0.39714 (11)	0.0374 (4)	
H5	0.6926	0.7844	0.4531	0.045*	
C6	0.64547 (12)	0.85086 (18)	0.34404 (11)	0.0362 (4)	
H6	0.6272	0.9310	0.3625	0.043*	
C7	0.63504 (11)	0.81283 (16)	0.26195 (11)	0.0299 (4)	
C8	0.60331 (11)	0.81418 (16)	0.12734 (11)	0.0306 (4)	
C9	0.57041 (13)	0.87542 (19)	0.04971 (12)	0.0399 (4)	
H9A	0.5808	0.8195	0.0032	0.060*	
H9B	0.6015	0.9545	0.0410	0.060*	
H9C	0.5076	0.8919	0.0547	0.060*	
C10	0.59373 (11)	0.45389 (15)	0.10668 (10)	0.0274 (3)	
C11	0.63578 (12)	0.33736 (17)	0.11793 (11)	0.0316 (4)	
H11	0.6971	0.3304	0.1102	0.038*	
C12	0.58623 (13)	0.23096 (17)	0.14081 (11)	0.0360 (4)	
H12	0.6134	0.1521	0.1493	0.043*	
C13	0.49582 (12)	0.24576 (16)	0.15059 (11)	0.0341 (4)	
C14	0.45240 (12)	0.36075 (18)	0.14095 (11)	0.0359 (4)	
H14	0.3912	0.3671	0.1494	0.043*	
C15	0.50226 (12)	0.46665 (16)	0.11837 (11)	0.0329 (4)	
H15	0.4748	0.5456	0.1111	0.040*	

### Atomic displacement parameters (Å^2^)

**Table:**

	*U*^11^	*U*^22^	*U*^33^	*U*^12^	*U*^13^	*U*^23^
S	0.0341 (2)	0.0313 (2)	0.0251 (2)	−0.00272 (17)	−0.00071 (16)	−0.00052 (16)
F	0.0562 (7)	0.0355 (6)	0.0558 (7)	−0.0171 (5)	−0.0002 (6)	0.0001 (5)
O1	0.0408 (7)	0.0264 (6)	0.0354 (6)	0.0025 (5)	−0.0010 (5)	0.0017 (5)
O2	0.0311 (6)	0.0435 (7)	0.0437 (7)	−0.0024 (5)	0.0071 (6)	−0.0052 (6)
C1	0.0280 (8)	0.0273 (8)	0.0276 (8)	−0.0023 (6)	−0.0021 (6)	0.0010 (6)
C2	0.0253 (8)	0.0270 (8)	0.0273 (8)	−0.0037 (6)	0.0007 (6)	0.0000 (6)
C3	0.0317 (9)	0.0301 (8)	0.0311 (9)	0.0002 (7)	−0.0003 (7)	0.0017 (7)
C4	0.0347 (9)	0.0397 (10)	0.0312 (9)	−0.0023 (7)	−0.0049 (7)	0.0057 (7)
C5	0.0409 (10)	0.0442 (10)	0.0270 (9)	−0.0082 (8)	−0.0001 (8)	−0.0041 (8)
C6	0.0422 (10)	0.0307 (9)	0.0357 (9)	−0.0039 (7)	0.0052 (8)	−0.0053 (7)
C7	0.0310 (9)	0.0266 (8)	0.0320 (9)	−0.0026 (6)	0.0005 (7)	0.0024 (7)
C8	0.0287 (8)	0.0294 (8)	0.0337 (9)	−0.0033 (7)	−0.0028 (7)	0.0019 (7)
C9	0.0414 (10)	0.0371 (10)	0.0412 (10)	0.0005 (8)	−0.0091 (8)	0.0097 (8)
C10	0.0311 (8)	0.0274 (8)	0.0236 (8)	−0.0021 (6)	−0.0033 (6)	−0.0037 (6)
C11	0.0306 (9)	0.0320 (9)	0.0324 (9)	0.0038 (7)	−0.0009 (7)	−0.0045 (7)
C12	0.0453 (10)	0.0265 (8)	0.0364 (9)	0.0037 (7)	−0.0036 (8)	−0.0042 (7)
C13	0.0422 (10)	0.0287 (8)	0.0314 (9)	−0.0099 (7)	−0.0028 (7)	−0.0040 (7)
C14	0.0276 (8)	0.0398 (10)	0.0402 (10)	−0.0024 (7)	−0.0023 (7)	−0.0042 (8)
C15	0.0304 (8)	0.0291 (9)	0.0393 (9)	0.0028 (7)	−0.0049 (7)	−0.0010 (7)

### Geometric parameters (Å, °)

**Table:**

S—O2	1.4953 (14)	C6—C7	1.382 (2)
S—C1	1.7525 (17)	C6—H6	0.9300
S—C10	1.7948 (17)	C8—C9	1.483 (2)
F—C13	1.357 (2)	C9—H9A	0.9600
O1—C8	1.371 (2)	C9—H9B	0.9600
O1—C7	1.382 (2)	C9—H9C	0.9600
C1—C8	1.358 (2)	C10—C11	1.385 (2)
C1—C2	1.452 (2)	C10—C15	1.390 (2)
C2—C3	1.393 (2)	C11—C12	1.388 (3)
C2—C7	1.393 (2)	C11—H11	0.9300
C3—C4	1.391 (2)	C12—C13	1.373 (3)
C3—H3	0.9300	C12—H12	0.9300
C4—C5	1.400 (3)	C13—C14	1.377 (3)
C4—H4	0.9300	C14—C15	1.385 (3)
C5—C6	1.382 (3)	C14—H14	0.9300
C5—H5	0.9300	C15—H15	0.9300
			
O2—S—C1	108.60 (8)	C1—C8—C9	132.78 (17)
O2—S—C10	106.21 (8)	O1—C8—C9	116.38 (15)
C1—S—C10	99.15 (8)	C8—C9—H9A	109.5
C8—O1—C7	106.58 (13)	C8—C9—H9B	109.5
C8—C1—C2	107.42 (15)	H9A—C9—H9B	109.5
C8—C1—S	122.38 (13)	C8—C9—H9C	109.5
C2—C1—S	130.04 (13)	H9A—C9—H9C	109.5
C3—C2—C7	119.34 (15)	H9B—C9—H9C	109.5
C3—C2—C1	136.18 (16)	C11—C10—C15	121.07 (16)
C7—C2—C1	104.45 (14)	C11—C10—S	118.27 (13)
C4—C3—C2	117.83 (16)	C15—C10—S	120.27 (13)
C4—C3—H3	121.1	C10—C11—C12	119.83 (16)
C2—C3—H3	121.1	C10—C11—H11	120.1
C3—C4—C5	121.28 (17)	C12—C11—H11	120.1
C3—C4—H4	119.4	C13—C12—C11	117.90 (16)
C5—C4—H4	119.4	C13—C12—H12	121.1
C6—C5—C4	121.55 (17)	C11—C12—H12	121.1
C6—C5—H5	119.2	F—C13—C12	118.42 (16)
C4—C5—H5	119.2	F—C13—C14	118.04 (16)
C5—C6—C7	116.21 (17)	C12—C13—C14	123.54 (16)
C5—C6—H6	121.9	C13—C14—C15	118.26 (16)
C7—C6—H6	121.9	C13—C14—H14	120.9
C6—C7—O1	125.51 (16)	C15—C14—H14	120.9
C6—C7—C2	123.78 (16)	C14—C15—C10	119.38 (16)
O1—C7—C2	110.70 (14)	C14—C15—H15	120.3
C1—C8—O1	110.84 (15)	C10—C15—H15	120.3
			
O2—S—C1—C8	125.82 (15)	C2—C1—C8—O1	−1.56 (19)
C10—S—C1—C8	−123.54 (15)	S—C1—C8—O1	−177.39 (11)
O2—S—C1—C2	−48.99 (17)	C2—C1—C8—C9	178.10 (18)
C10—S—C1—C2	61.65 (16)	S—C1—C8—C9	2.3 (3)
C8—C1—C2—C3	−176.81 (19)	C7—O1—C8—C1	1.47 (18)
S—C1—C2—C3	−1.4 (3)	C7—O1—C8—C9	−178.25 (15)
C8—C1—C2—C7	1.00 (18)	O2—S—C10—C11	−12.66 (15)
S—C1—C2—C7	176.41 (13)	C1—S—C10—C11	−125.19 (13)
C7—C2—C3—C4	1.4 (2)	O2—S—C10—C15	174.41 (13)
C1—C2—C3—C4	178.99 (18)	C1—S—C10—C15	61.88 (15)
C2—C3—C4—C5	−1.0 (3)	C15—C10—C11—C12	0.3 (3)
C3—C4—C5—C6	0.1 (3)	S—C10—C11—C12	−172.52 (13)
C4—C5—C6—C7	0.4 (3)	C10—C11—C12—C13	0.7 (3)
C5—C6—C7—O1	−178.95 (16)	C11—C12—C13—F	177.80 (15)
C5—C6—C7—C2	0.1 (3)	C11—C12—C13—C14	−1.7 (3)
C8—O1—C7—C6	178.34 (17)	F—C13—C14—C15	−177.99 (15)
C8—O1—C7—C2	−0.79 (18)	C12—C13—C14—C15	1.5 (3)
C3—C2—C7—C6	−1.0 (3)	C13—C14—C15—C10	−0.3 (3)
C1—C2—C7—C6	−179.27 (16)	C11—C10—C15—C14	−0.6 (3)
C3—C2—C7—O1	178.14 (14)	S—C10—C15—C14	172.18 (13)
C1—C2—C7—O1	−0.12 (18)		

### Hydrogen-bond geometry (Å, °)

**Table:**

*D*—H···*A*	*D*—H	H···*A*	*D*···*A*	*D*—H···*A*
C6—H6···F^i^	0.93	2.52	3.346 (2)	148
C9—H9B···O2^ii^	0.96	2.41	3.189 (2)	138

Symmetry codes: (i) −*x*+1, *y*+1, −*z*+1/2; (ii) −*x*+3/2, *y*+1/2, *z*.
